# Advances of Stem Cell-Laden Hydrogels With Biomimetic Microenvironment for Osteochondral Repair

**DOI:** 10.3389/fbioe.2020.00247

**Published:** 2020-03-31

**Authors:** Bingbing Xu, Jing Ye, Fu-Zhen Yuan, Ji-Ying Zhang, You-Rong Chen, Bao-Shi Fan, Dong Jiang, Wen-Bo Jiang, Xing Wang, Jia-Kuo Yu

**Affiliations:** ^1^Knee Surgery Department of the Institute of Sports Medicine, Peking University Third Hospital, Beijing, China; ^2^School of Clinical Medicine, Weifang Medical University, Weifang, China; ^3^Clinical Translational R&D Center of 3D Printing Technology, Shanghai Ninth People's Hospital, Shanghai Jiao Tong University School of Medicine, Shanghai, China; ^4^Beijing National Laboratory for Molecular Sciences, State Key Laboratory of Polymer Physics & Chemistry, Institute of Chemistry, Chinese Academy of Sciences, Beijing, China; ^5^University of Chinese Academy of Sciences, Beijing, China

**Keywords:** stem cell-laden hydrogels, microenvironment, extracellular matrix, osteochondral tissue engineering, regenerative medicine

## Abstract

Osteochondral damage from trauma or osteoarthritis is a general joint disease that can lead to an increased social and economic burden in the modern society. The inefficiency of osteochondral defects is mainly due to the absence of suitable tissue-engineered substrates promoting tissue regeneration and replacing damaged areas. The hydrogels are becoming a promising kind of biomaterials for tissue regeneration. The biomimetic hydrogel microenvironment can be tightly controlled by modulating a number of biophysical and biochemical properties, including matrix mechanics, degradation, microstructure, cell adhesion, and intercellular interactions. In particular, advances in stem cell-laden hydrogels have offered new ideas for the cell therapy and osteochondral repair. Herein, the aim of this review is to underpin the importance of stem cell-laden hydrogels on promoting the development of osteochondral regeneration, especially in the field of manipulation of biomimetic microenvironment and utilization growth factors with various delivery methods.

## Introduction

Osteochondral interface defects generally involve lesions in articular cartilage and subchondral cartilage. Cartilage is essentially avascular and less cellular, and lacks the ability to repair itself (Abdel-Sayed and Pioletti, 2015). Meanwhile, if cartilage defects are not treated, joints will gradually and irrevocably deteriorate, leading to severe osteoarthritis and eventually disability (Chen et al., 2011). Current treatment strategies for osteochondral defects mainly include the microfracture (bone marrow stimulation) (Dasar et al., 2016), auto-transplantation and allografts of osteochondral (VanTienderen et al., 2017), and autologous chondrocyte implantation (Beck et al., 2018). Despite their widespread usage in the actual clinic, there are still obvious and inevitable limitations and shortcomings. For example, microfracture treatment may cause the formation of fibrocartilage with poor biological function (Steinwachs and Kreuz, 2007; Becher et al., 2019). Autologous chondrocyte implantation has been applied for 20 years in clinic, but there are still disadvantages like shortage source and long harvest time of chondrocyte, periosteal hypertrophy and ablation (Lohan et al., 2013), and low effectiveness for elderly patients (Giannoni et al., 2005). Allografts are plagued by limited supply, immune rejection, insufficient integration, and low cell viability. Autologous transplantation lacks integration and tissue sources, and requires additional surgery that may induce the potential disease at the donor site (Bal et al., 2010; Sartori et al., 2017). Compared to the above-mentioned strategies, osteochondral tissue engineering has been proposed and approved for more effective treatment, among which the stem cell research has been of great importance in the biomedical and tissue regenerations.

Tissue engineering, consisting of scaffolds, cells and favorable growth factors, has evolved into the most promising therapeutic strategy for cartilage tissue reconstruction (Huang et al., 2018; Wang et al., 2018). In order to achieve perfect regeneration of damaged cartilage, biodegradable scaffolds must be provided to simulate local characteristics of specific tissues, transport growth factors and tissue cells for newly formed tissues (Polo-Corrales et al., 2014). In the best case, cartilage tissue-engineered scaffolds should be characterized by porous, non-toxic, biodegradable, biocompatible, and promoted cell differentiation and tissue regeneration. In order to construct an ideal tissue-engineering program, it is important to design a functional biomaterial that essentially mimics the natural extracellular matrix (ECM) component of cartilage. Traditional methods typically include the precise incorporation of bioactive growth factors into target tissue, the use of cell-free scaffold biomaterials, and mimic natural ECM with the use of cell-laden building scaffolds, especially for three-dimensional (3D) porous scaffolds, which are the most commonly used biomaterials to facilitate cell organization into ECM during reconstruction (Ansboro et al., 2014; Du et al., 2014; Bernhard and Vunjak-Novakovic, 2016).

As a most promise of future tissue engineering and regeneration, stem cells with multidirectional differentiation potentials can be used to promote tissue growth, metabolism, repair and microenvironmental stability. Stem cells are characterized by their ability to self-renew and differentiate into various mature cells, which have inspired the development of biomedical science (Madl and Heilshorn, 2018), including the applications of regenerative medicine methods, repair or replacement of damaged tissues, disease modeling, and pharmacology screening platforms. However, simulating the unique biological functions of articular cartilage remains a challenge, because the composition and regional structure of these joints is highly complex. Tissue engineering methods offer appropriate biomaterials as artificial ECM to promote stem cell growth, proliferation and differentiation at defect sites, leaving the regeneration of articular cartilage to the involved natural biological processes that stem cells can interact with soluble factors. Stem cells reside in a specialized microenvironment *in vivo*, called the stem cell niche which is both dynamic and complex (Li and Xie, 2005; McClenahan et al., 2016). Biophysical and biochemical factors form the niche that guides the fate of resident stem cells. Many of these factors are provided by the microstructure, biochemical composition and mechanical properties of the ECM. In the field of tissue engineering, it is a popular strategy to control engineered niches by using the characteristics of scaffold materials to guide the differentiation and maturation of stem cells into functional tissue constructs.

Hydrogels are consisting of natural or synthetic hydrophilic polymer chains connected to each other at the crosslinking point, which have a unique 3D crosslinked polymer network covering a wide range of chemical compositions and physical properties (Paschos et al., 2015; Liu et al., 2016). The natural hydrophilicity of polymer chains enables hydrogels to absorb a certain amount of water and be applied in various technical biomaterials for drug delivery and tissue regeneration. Especially, *in situ* hydrogels have the advantages of simple drug preparation and strong ability to deliver drugs, peptides and cells. Hydrogels have a unique combination similar to natural ECM and are attractive biomaterials for the osteochondral tissue engineering. The hydrogel microenvironment can be strictly controlled through the adjustment of many biophysical and biochemical properties, such as the matrix mechanics, degradability, microstructure, cell adhesion, and cell-cell interactions (Brown and Anseth, 2017; Jekhmane et al., 2019). These properties can be easily manipulated to suit for a variety of biomedical applications (Sun et al., 2018). Therefore, stem cell-hydrogel constructs could be personalized for patients using the advanced technology. Hydrogels that combine stem cells and growth factors have great potential to challenge regeneration of osteochondral defects. In the past decade, basic research on osteochondral tissue engineering of stem cell-laden hydrogels systems with biomimetic microenvironment has achieved remarkable success, bringing promise for osteochondral tissue repair (Li et al., 2018; Xu et al., 2019).

This review will focus on the importance and development of biomimetic microenvironment using the engineering cell-laden hydrogels on promotion of osteochondral tissue engineering and regeneration medicine fields, mainly including extracellular matrix, engineered matrix degradation, microarchitecture, cell-adhesive ligands, and cell-cell interactions. We also summarize the strategies for repairing cartilage defects by stem cell-laden hydrogels and discuss how various growth factors and delivery methods affect stemness maintenance and differentiation to facilitate the chondrogenesis or osteogenesis within the hydrogels. Finally, we provide some suggestions and prospects on developing stem cell-laden hydrogels via tailoring of their biomimetic microenvironment (e.g., physicochemical and mechanical properties) for effective osteochondral tissue engineering. Understanding medical needs and concurrently lessening the difficulty of hydrogel construction should therefore be the goal for future research in regeneration medicine fields.

## Effects of Biomimetic Microenvironment on the Engineering Hydrogels

The stem cell niche consists of a myriad of interacting ECM components, which can provide many biophysical and biochemical inputs to regulate the stem cell functions such as cell populations, self-recovery, quiescence, differentiation, etc. (Xie and Spradling, 2000). The most important factors are the interactions among the stem cells, neighboring differentiated cells and ECM (Morrison et al., 1997). Additionally, other factors like oxygen level, ion concentration, growth factors, and cytokines also play important roles (Drueke, 2006; Scadden, 2006; Hsu and Drummond-Barbosa, 2009; Eliasson and Jonsson, 2010). In this section, we will focus on the effects of matrix mechanics, on-demand degradation, microstructure, cell-adhesive ligands and cell-cell interactions for maintaining and regulating stem cells in the engineering hydrogels (Fuchs et al., 2004).

### Extracellular Mechanics

ECM, mainly including geometry, elasticity and mechanical signals, provides the necessary stimuli to control the shape, activity, and migration of stem cell (Lv et al., 2015). Especially, mechanical forces from the ECM and subsequent alterations in intracellular tension can regulate stem cell differentiation via the cytoskeletal tension and RhoA-ROCK pathway activation (Shah et al., 2014). As for the tissue engineering, extracellular mechanics like stiffness and viscoelasticity play important roles in the signal pathways between cells to tailor the stem cell proliferation behaviors and regenerative qualities (Hoben et al., 2008; Chang and Knothe Tate, 2011).

#### Extracellular Stiffness

**Figure 1:**
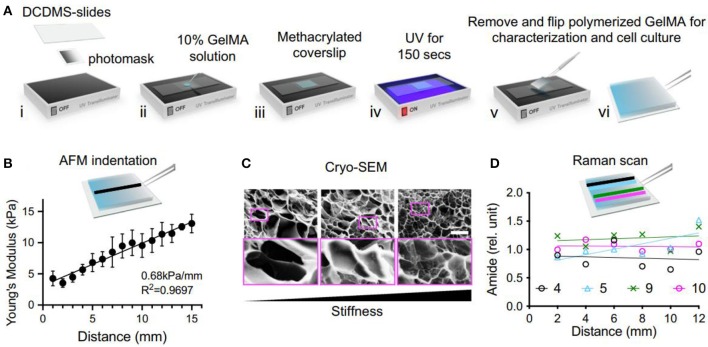
The fabrication and characterization of GelMA stiffness gradient hydrogel. **(A)** Schematic of UV polymerization. **(B)** Atomic force microscopy (AFM) measurement. **(C)** Images from cryo-Scanning electron microscopy (SEM) showed an inverse correlation to stiffness. **(D)** Raman scanning does not show a significant change in amide amount on the stiffness gradient. Reproduced with permission from Kim et al. (2020).

Stiffness is typically described by an elastic or Young's modulus, which is defined as the ratio of applied stress (i.e., force per area) to strain (i.e., relative deformation) for small perturbations. ECM can be recognized as a cross-linked polymer network, possessing the time-independent stiffness behavior. This mechano-sensing ability can affect the fundamental cellular functions. With this understanding, development of stiffness hydrogels is useful for researching the mechanical interactions between stem cells and extracellular environments. For example, Kim et al. developed a linear stiffness gradient hydrogel via tailoring the polymerization of gelatin methacryloyl (GelMA) with a gradient UV photomask for stem cell mechano-sensation and differentiation abilities (Figure 1; Kim et al., 2020). Furthermore, they also found human adipose-derived stem cells (hADSCs) could increase chondrogenic roles *in vivo* by controlling the stiffness of cell-free and cell-embedded fibrin hydrogel; in this case, optimal scaffolds could promote both cell survival and chondrogenic potential for cartilage tissue engineering (Jung et al., 2010).

#### Extracellular Viscoelasticity

**Figure 2:**
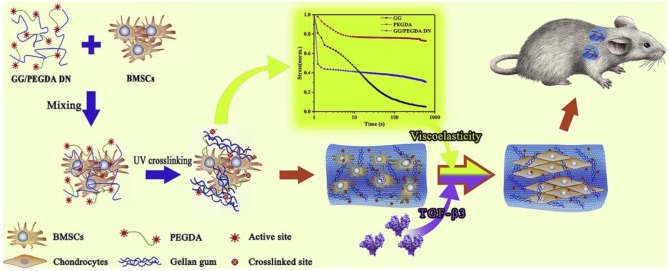
Schematic illustration of experimental approach of bone marrow-derived mesenchymal stem cells (BM-MSCs) encapsulated in GG/PEGDA DN hydrogels and chondrogenic differentiation *in vivo*. Reproduced with permission from Li et al. (2020).

Besides for the commonly used elastic hydrogel systems to tailor stem cell mechanobiology and activity, natural ECM components are also viscoelastic materials with stress-relaxation behavior (Levental et al., 2007; Geerligs et al., 2008). Hydrogels composed of reconstituted ECM proteins like collagen and fibrin exhibit stress relaxation in response to a constant load pressure (Isono and Nishitake, 1995), because polymer chains within the network can rearrange in order to dissipate the applied force from the molecular level. Thus, recent efforts have been directed toward designing hydrogels with tuneable viscoelasticity to recapitulate the ECM and cell interactions (Haugh and Heilshorn, 2016). Li et al. synthesized a kind of GG/PEGDA DN hydrogel through the linkage of gellan gum (GG) with polyethylene glycol diacrylate (PEGDA) to offer physical environment for mesenchymal stem cells (MSCs) proliferation, spreading, chondrogenic differentiation and cartilage tissue engineering (Figure 2; Li et al., 2020). Xie et al. also found that viscoelasticity played a significant role in expanding seed cells for articular cartilage tissue engineering and regeneration (Xie et al., 2019).

### Matrix Degradation

Matrix degradation of the ECM affects stem cell proliferation, self-recovery, quiescence and differentiation through the integrins (Daley et al., 2008), because the cell-secreted enzymes can degrade the native ECM to upgrade the cell spreading and migration capacities through the matrix. By mimicking this dynamic matrix degradation for 3D cell culture, engineered hydrogel had been well-developed by passive hydrolysis of ester crosslinkers for polyethylene glycol (PEG) hydrogels (Sawhney et al., 1994; Bryant et al., 2004). In addition, introduction of enzyme can significantly promote the degradation behaviors to permit cell-mediated remodeling. Lutolf et al. prepared a kind of PEG hydrogels with peptides susceptible to cleavage by matrix metalloproteinases (MMPs). Altering the amino acid sequence of peptide cause the various affinity of MMPs for the peptides, thus controlling the hydrogel degradation kinetics (Park et al., 2004). Kloxin et al. developed a photodegradable PEG hydrogel system that possessed the selective degradation behavior with spatial and temporal material resolution; in this case, mechanics, mesh size and swelling property of hydrogels were necessarily tailored similar to modulating 3D stiffness methods (Kloxin et al., 2009).

### Microarchitecture

The innate hierarchical structure and composition distribution of the musculoskeletal tissue interface is destroyed and replaced by fibrotic tissue in the case of disease and degeneration. The focus of the tissue-engineering strategy is to restore the transitional complexity found at the junction of regenerative medicine. For bio-mimicking the 3D contexts, strategies for generating biomimetic fibrous topographies are urgently needed by designing the substrates with well-defined engineered features like grooves, pits and pillars with the order of hundreds of nanometers to tens of micrometers (Lu et al., 2016). Common techniques for producing macroporous hydrogels contain microparticle template, freeze dry, and gas foam (Kuo and Ma, 2011), which represents a class of 3D materials with engineered topographical variation. In addition, cross-linking of hydrogel microribbons (Han et al., 2013) and self-assembly of microgels (Griffin et al., 2015) have also been recognized as alternative techniques to gain architecture hydrogels. Due to limitations in material fabrication techniques, pore size is known as a key factor for cells to interact with the implanted hydrogel materials, thereby 3D architecture can modify the transport properties of cellular microenvironments and endow an additional mechanism to modulate the stem cell microenvironment (Wolf et al., 2014). For example, Zhu et al. constructed an injectable continuous stratified scaffold and designed multiple cell systems to enhance the osteochondral regeneration. The biomimetic constructs of structure and function not only stimulated the regeneration of hyalcartilage and subchondral bone, but also promoted integration of newly formed tissue with the host tissue (Zhu et al., 2019).

### Cell-Adhesive Ligands

**Figure 3:**
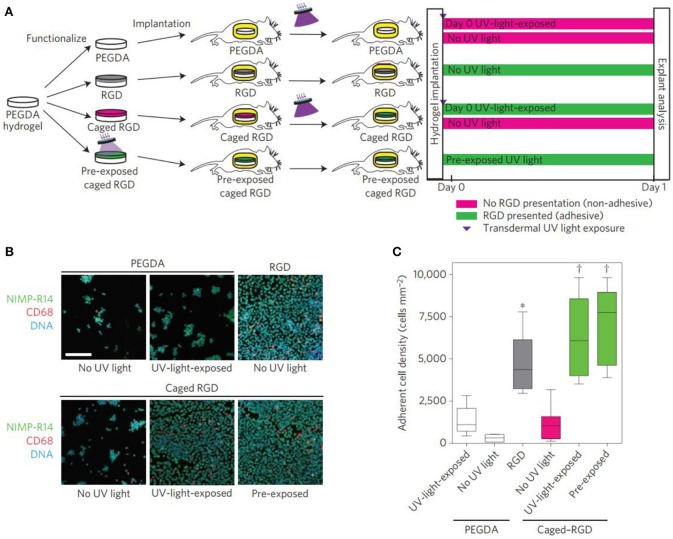
Transdermal activation of *in vivo* inflammatory cell adhesion. **(A)** Schematic representation of the time line for *in vivo* activation of cell adhesion using transdermal UV light exposure. **(B)** Photographs of explanted hydrogels stained for adherent inflammatory cells (green, NIMP-R14 (neutrophil); magenta, CD68 (macrophage); blue, DAPI (DNA); scale bar = 80 μm). Reproduced with permission from Lee et al. (2015). **(C)** Adherent cell density, box-whisker plot for 6–8 mice per group, demonstrating light-based triggering of inflammatory cell adhesion to caged RGD-presenting implants. ANOVA *p* < 0.0001, ***p < 0.05 vs. UV-light-exposed PEGDA, †*p* < 0.001 vs. no-UV-light caged RGD.

Specific cell-matrix adhesion is required for cell spreading, migration and mechano-sensing via cell surface receptors. In particular, a class of heterodimeric receptors as integrins link the intracellular cytoskeleton to the specific cell-adhesive ligands on ECM proteins (Barczyk et al., 2010). Tripeptide arginine-glycine-aspartic acid (RGD) is found in multiple ECM proteins and binds to several different integrin dimers, which facilitates cell spreading and migration (Cao et al., 2016) and has been incorporated into hydrogel systems to construct the adhesion of various cell types (Hersel et al., 2003; Cipriani et al., 2019). Besides, combinations of other ligands with RGD are also necessary to elicit the desired behaviors. In order to solve the difficulty in the ligand interactions, ligand concentrations, identity and nanoscale spacing of cell-adhesive ligands should be optimized to regulate the cell activity (Jongpaiboonkit et al., 2008; Lam et al., 2015). Therefore, strategies to pattern adhesive ligands in hydrogels have been developed to control cellular access to adhesive cues by cell-matrix adhesion (Luo and Shoichet, 2004; Ekerdt et al., 2013). An alternative strategy had been to incorporate photocaged adhesive peptides into the hydrogels that were initially inaccessible for cell binding (Wirkner et al., 2011). In these systems, many photochemical approaches to hydrogel modification above have been employed to temporally control ligand availability (Figure 3; Lee et al., 2015). Kloxin et al. used the photoreactive methods to control the hydrogel degradation for selectively releasing the RGD peptides and decreasing the adhesivity after a defined culture interval (Kloxin et al., 2009). Boekhoven et al. applied the host-guest interactions to display RGD peptides from alginate surfaces. Surfaces initially presenting cell-adhesive RGD peptides could be rendered non-adhesive by addition of a control peptide with a stronger host-guest binding partner (Boekhoven et al., 2013). Other self-assembly approaches have also been employed complementary leucine zipper peptides (Liu et al., 2010) and complementary DNA strands to achieve dynamic control over ligand presentation dynamics (Zhang et al., 2013).

### Cell-Cell Interactions

**Figure 4:**
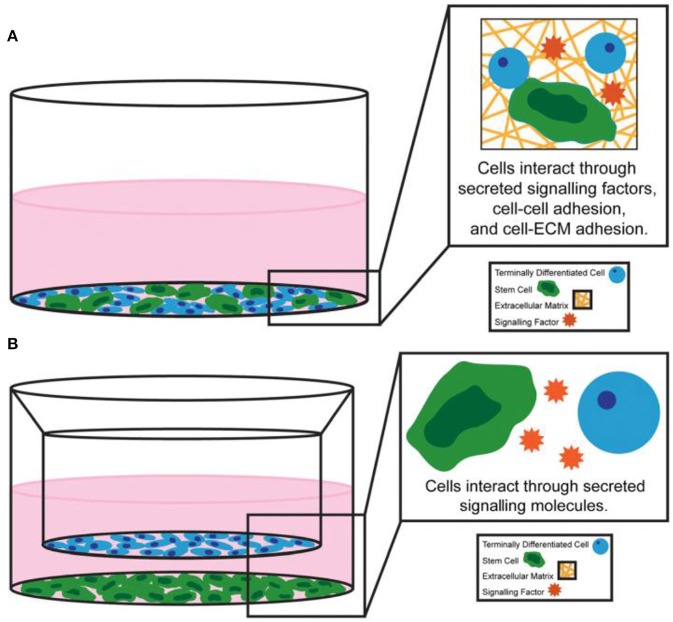
Examples of **(A)** direct co-culture system and **(B)** indirect co-culture system. Reproduced with permission from Paschos et al. (2015).

Differentiated progeny and heterologous cells communicate with direct cell-cell contact and the secretion of soluble factors, thus continuously exchanging signals related to stem cell fate, activity and differentiation. Among them, direct cell-cell contact was usually achieved through three main types of cellular junctions: gap junctions, tight junctions and adherent junctions (Figure 4A; Paschos et al., 2015). Optimized co-culture of stem cells with other cell types allows stem cells to remain pluripotent or trigger differentiation into the desired lineage (Kaji et al., 2011; Paschos et al., 2015).

The natural ECM components contain the binding motifs of various soluble signals, for instance, the growth factors. These natural interactions inspired the desired engineering bioactivity of artificial stem cell niches. Earlier studies have shown that growth factors immobilized on solid substrates kept the biological activity of stem cells (Kaji et al., 2011) and more effective than their soluble counterparts in some cases (Tamama et al., 2006). It is more biomimetic for localizing cell-secreted factors to incorporate charged polysaccharides to sequester growth factors (Hortensius and Harley, 2013) or peptide sequences binding the secreted ECM proteins (Cook et al., 2017). Instead, growth factors are designed to increase the effectiveness of these factors by increasing their longer-term interaction with natural ECM (Martino et al., 2014). Many biological processes, e.g., cell migration and tissue morphogenesis, are more sensitive to the concentration gradient of soluble factors than to their uniform expression. Various engineering strategies have been applied to generate gradients in hydrogel systems, such as microfluidic devices and spatial patterns of growth factor-chelating molecules (Kim et al., 2014).

In addition, stem cells can interact with other niche cells by the indirect cell-cell contact (Figure 4B; Paschos et al., 2015). Cell adhesion molecules were immobilized to the surface by fusing immunoglobulin Fc domain of the e-cadherin cell outfield in the early engineering system experiments (Miki et al., 2008). Recently, HAVDI peptides have been conjugated to hyaluronic acid (HA) hydrogels through the mediation of n-cadherin (Nagaoka et al., 2002). In addition to cadherin-mediated contact, peptide sequences that simulate the activity of neural cell adhesion molecule were incorporated into engineered elastin like protein materials (You et al., 2015).

## Biological Regulatory Factors for Chondrogenesis and their Delivery Methods of MSCs-Laden Hydrogels

### Biological Regulatory Factors for Chondrogenesis

**Figure 5:**
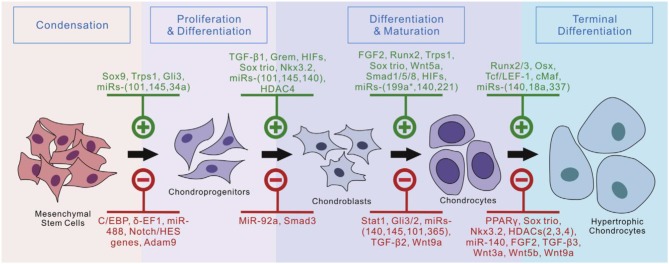
Currently identified regulators of chondrogenic differentiation from MSCs. Reproduced with permission from Green et al. (2015).

The native ECMs could separate biological regulatory factors for promoting cell proliferation and differentiation. These biological regulatory factors include multiple signaling pathways, including transforming growth factor beta (TGF-β)/bone morphogenic proteins (BMPs), fibroblast growth factors (FGFs), hedgehog, notch, Wnt/β-catenin, angiogenic, and hypoxia signaling pathways. Many biomodulators of chondrogenesis provide multiple methods to induce chondrogenic formation and differentiation in MSCs (Figure 5; Green et al., 2015). Current research indicates that TGF-β proteins were the most effective inducers of chondrogenesis in human mesenchymal stem cells (hMSCs) among regulatory factors involved in regulating chondrogenesis (Stevens et al., 2004; Jin et al., 2007; Zhao and Hantash, 2011).

### Delivery of Biological Regulatory Factors

There are five ways to deliver biological regulators: freeform in medium, physical mixing in hydrogel, microencapsulation, covalent bond with hydrogel, and gene delivery.

#### Freeform in Medium

Free-form biomodulator delivery in culture medium is an effective method for culture of engineered cell-laden hydrogel osteochondral constructs *in vitro*. During the monolayer expansion process before hydrogel encapsulation, it was found that MSCs exposed to TGF-β3 in culture medium can form chondrocyte populations of different maturities at 7 and 14 days (Lam et al., 2014). The specially designed two-chamber well-provides both osteogenic and chondrogenic stimulation by freeform biomodulator in medium to rabbit BM-MSCs located in different areas of the scaffold (Chen et al., 2016). However, the need for frequent administration to maintain the concentration and biological activity of biological regulators in the medium is not best for the long-term culture of tissue-engineering.

#### Physical Mixing in Hydrogel

Encapsulating biological regulatory factors into the hydrogels is a simple and efficacious way for sustained release. BMP-2 can be delivered through MMP-based sensitive hydrogels to promote osteochondral repair *in vivo* (Holloway et al., 2014). Oligo [poly(ethylene glycol) fumarate] hydrogel composites containing TGF-β1-loaded gelatin microparticles and MSCs were implanted in osteochondral defects and facilitated subchondral bone formation (Guo et al., 2010). α2β1 integrin-specific peptide (GFOGER)-functionalized hydrogels with MSCs can continuously release low doses of BMP-2 around the periphery and enhance bone repair capabilities (Shekaran et al., 2014). Delivering biological regulators through encapsulation in hydrogels may require no multiple dosages and may keep release for several weeks, which was beneficial for osteochondral tissue engineering. And the delivery could apply to the formation of cartilage *in vivo*. However, the efficiency of chondrogenic induction may be limited by the fact that the amount of biological regulator from hydrogels significantly decreases over time.

#### Microencapsulation

**Figure 6:**
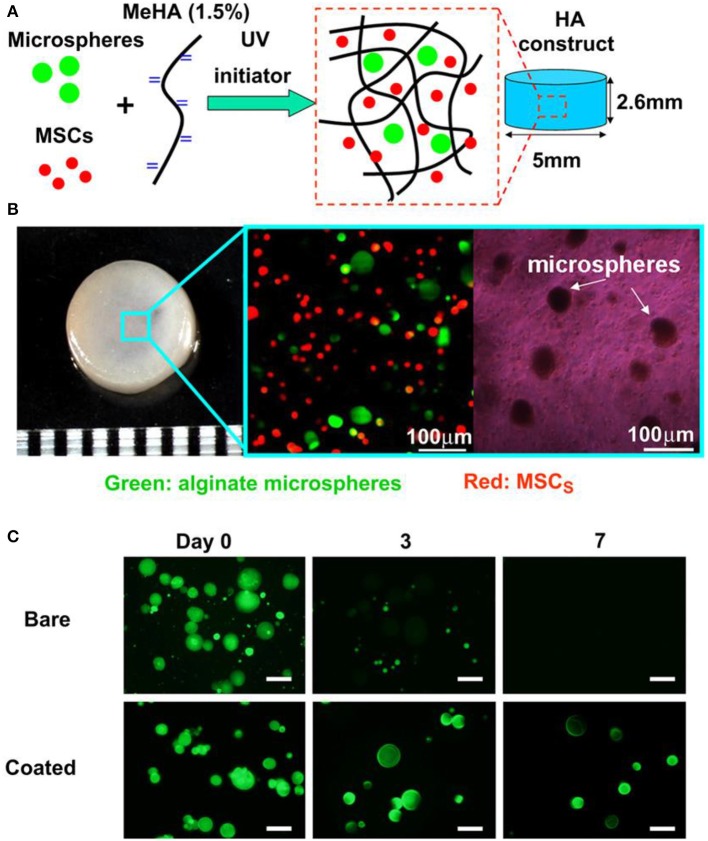
Microencapsulation preparation and *in vitro* culture. Photoencapsulation of alginate microspheres and MSCs into Methacrylated HA (MeHA) hydrogel disks **(A)**; fabricated HA hydrogel disk and fluorescent and bright field microscopic images of MSCs (membrane labeled with red dye) and alginate microspheres (containing FITC-labeled protein) encapsulated in HA gels **(B)**; release of encapsulated BSA-FITC from bare and coated alginate microspheres over 7 days in PBS **(C)**, scale bar = 50 μm. Reproduced with permission from Bian et al. (2011).

The controlled local delivery of growth factors is another applicable strategy for cultivating engineered osteochondral constructs by MSCs. For this purpose, chondrocyte cells growth factors could be loaded into microencapsulation and further embedded in different regions of the constructs (Kim et al., 2016). In order to investigate the use of transplantable constructs for cartilage repair, Bian et al. studied the co-encapsulation of TGF-β3 containing alginate microspheres and hMSCs in HA hydrogels. HA hydrogel constructs inoculated with MSCs and microspheres containing TGF-β3 had comparable mechanical properties and cartilage matrix content compared to those continuously added TGF-β3 in the medium, while those directly encapsulated in a gel containing no microspheres had poor performance (Figure 6; Bian et al., 2011). The constructs including TGF-β3 microspheres also formed excellent cartilage matrix after implanted subcutaneously in nude mice. Moshaverinia et al. develop a novel co-delivery system based on TGF-β1 loaded RGD-coupled alginate microspheres encapsulating dental MSCs. And ectopic cartilage tissue regeneration has been observed inside and around the transplanted microspheres in animal studies (Moshaverinia et al., 2013). Although this approach is very useful to control release speed and improve delivery efficiency, the process may add complexity to the preparation and design of scaffolds.

#### Covalent Bond With Hydrogel

Hydrogel systems allow chelating biological regulatory factors through covalent binding, which has advantages over other delivery ways. Since the diffusion of small molecular weight proteins in hydrogels is very rapid, the strategy of immobilizing growth factors in the bioactive, physiologically related hydrogel-microenvironment is an important step in guiding cells to regenerate cartilage tissue. Benoit et al. first controlled the induction of multiple hMSC lineages purely by interacting with small molecular chemical functional groups bound to the hydrogel materials (Benoit et al., 2008). The proliferation of encapsulated cells and the production of cartilage ECM were increased by immobilizing TGF-β1 to thiol-ene PEG hydrogel by covalent bounds over a period of 28 day (Sridhar et al., 2014), which levels exceeded those of cells in hydrogels in culture medium with dosed TGF-β1 or untreated. Such growth factor delivery methods by simple chemistry to control high levels of cell proliferation and differentiation would be particularly powerful because they are simpler, cheaper and easier to control.

#### Gene Delivery

Integrating therapeutic genes into biomaterials is a new method of delivering regulatory factors that promote tissue regeneration. Non-viral gene therapy may provide more physiological, long-lasting and cost-effective alternatives (Meinel et al., 2006). Compared with pre-synthesized recombinant proteins, the expression of gene products guarantees true post-translational modification, which reduced possible immunogenicity and increased the biological activity. There are two kinds of gene delivery vehicles: viral vectors and non-viral vectors. Viral vectors have a highly evolved mechanism for delivering DNA to cells, but could induce an effective immune response in host cells (Franceschi et al., 2004). Non-viral vector delivery is a promising gene therapy way, including cationic polymers, cationic polypeptides, and cationic liposomes (Yang et al., 2018). In tissue engineering applications, gene therapy can be combined with biomaterials to extend, persist and locally deliver the target protein *in situ*. Tomas et al. have developed and identified a novel nanohydroxyapatite (nHA)-mediated plasmid DNA (pDNA) encoding-activated alginate hydrogel that can direct the fate of MSCS toward either a chondrogenic or osteogenic phenotype by delivering TGF-β3 and/or BMP-2 (Gonzalez-Fernandez et al., 2016), which may be important to the clinical treatment of osteochondral defects. Non-viral dual delivery of VEGF and BMP2 in a collagen-nanohydroxyapatite scaffold accelerates the bone regeneration of MSCs *in vitro* and vascularization and bone repair by host cells *in vivo* (Curtin et al., 2015). However, compared to viral vectors, non-viral vectors are often disregarded due to their relatively inefficient.

## Cartilage Defects Repair of Stem Cell-Laden Hydrogels

The production of functional substitutes for autogenous cartilage and the development of new therapeutic strategies for cartilage defects are significant challenges that can be addressed through the field of tissue engineering. MSCs have become the most widely used stem cell in regenerative medicine due to their abundant cell sources, low immunogenicity, no ethical issues and minimal risk of teratomas (Wang et al., 2016). Cell therapy and tissue engineering have been combined in the repair of cartilage defects. MSC has the ability to multidirectionally differentiate into a variety of cells, including the chondrogenesis. Treatment combining MSCs and hydrogel are being applied in cartilage tissue engineering. The “medical signaling cell” properties associated with their immunomodulatory and anti-inflammatory effects induce the establishment of regenerative microenvironments in injured tissues. These nutritional effects, along with the long-established cartilage generator capacity, can be used for tissue-engineered constructs for articular cartilage repair. This section will focus on the cell therapy and tissue engineering of various MSCs for articular cartilage damage.

### Bone Marrow-Derived Mesenchymal Stem Cells (BM-MSCs)

**Figure 7:**
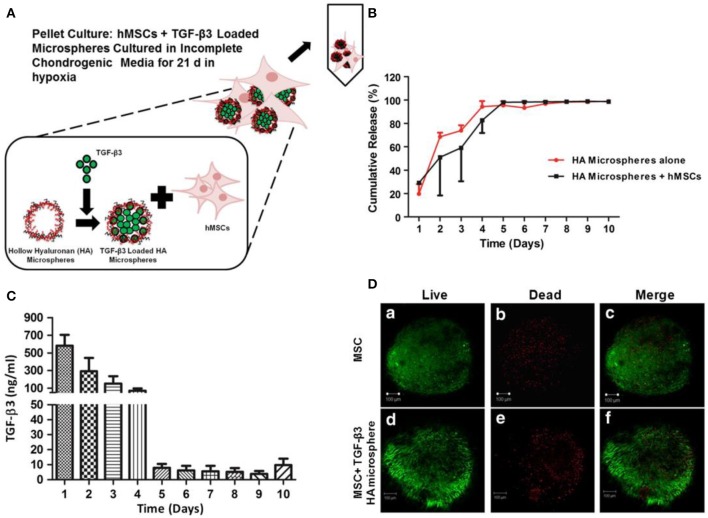
Loading, viability, and release of TGF-β3 from hollow HA microspheres. **(A)** An illustration of an HA microsphere pellet culture system loading TGF-β3. **(B)** Cumulative release profile of TGF-β3 from microspheres with/without hMSCs. **(C)** The release of TGF-β3 from HA microspheres cultured with hMSCs. **(D)** Viability of chondrogenic pellets cultured for 21 d with (d–f) and without (a–c) microspheres loading TGF-β3. Scale bar = 100 μm. Reproduced with permission from Ansboro et al. (2014).

BM-MSCs reside in bone marrow have been widely used in animal models and some clinical cases to study their chondrogenic potential for the treatment of OA (Zhang et al., 2019). Erickson et al. studied that the pre-maturation of MSC-seeded HA hydrogels *in vitro* could improve cartilage repair (Erickson et al., 2012). Vishal et al. studied MSC-seeded HA neocartilage and anatomic MSC-seeded HA constructs crosslinked by ammonium persulfate and N,N,N'N'-tetramethylethylenediamine for hMSC chondrogenesis in chondral defects (Figure 7; Ansboro et al., 2014). Meng et al. designed a composite scaffold combining affinity peptide-modified demineralized bone matrix particles with chitosan hydrogels for cartilage engineering, exhibiting appropriate porosity and providing a microenvironment for cell adhesion and proliferation. The functional composite of demineralized bone matrix particles and chitosan hydrogels (DBM-E7/CS) scaffold increased matrix production and improved the cartilage differentiation ability of BM-MSCs *in vitro*, which was a choice for repairing irregularly shaped cartilage defects (Meng et al., 2015). In clinical cases, the use of BM-MSCs in cartilage repair circumvented limitations of autologous chondrocyte implantation (ACI). BM-MSCs implantation for cure of cartilage defects achieved the equivalent clinical results as the first-generation ACI over a period of up to 10 years, with no significant increased risk of tumor formation (Teo et al., 2019).

### Adipose-Derived Stem Cells (ADSCs)

Adipose tissue as a rich source of MSCs has aroused great interest in cartilage tissue engineering. ADSCs are easily obtained in high yields through minimally invasive surgery (such as liposuction) (Tapp et al., 2008). Popa et al. proposed κ-carrageenan as a potential hydrogel that can be used for cell transport and for further application in cartilage regeneration. ADSCs encapsulated in κ-carrageenan hydrogel can still survive, proliferate and differentiate into cartilage cells (Popa et al., 2013). Rizk et al. evaluated that TGF-β1-fixed scaffolds prepared by incorporating TGF-β1-loaded gelatin microspheres into the poly(lactic-co-glycolic acid) (PLGA) framework enhanced the differentiation of ADSCs into chondrocytes (Yin et al., 2015). Furthermore, hADSCs can be mixed with sodium alginate and gelatin, combination with 3D bioprinting technology, to form a 3D bioprinted body of hADSCs-sodium alginate-gelatin mixture, which had the ability of ectopic bone formation in nude mice (Song et al., 2016). Huang et al. showed that the biomimetic matrix from chitosan-HA provided a suitable environment to support the differentiation of chondrocytes from ADSCs to the cartilage matrix (Huang et al., 2019). Fan et al. corroborated that an injectable bioorthogonal dextran-based hydrogel can support the chondrogenesis of ADSCs *in vitro* and *in vivo*, highlighting the role of bioorthogonal hydrogels for stem cell-based cartilage regeneration (Fan et al., 2018).

### Umbilical Cord Blood-Derived Mesenchymal Stem Cells (UCB-MSCs)

**Figure 8:**
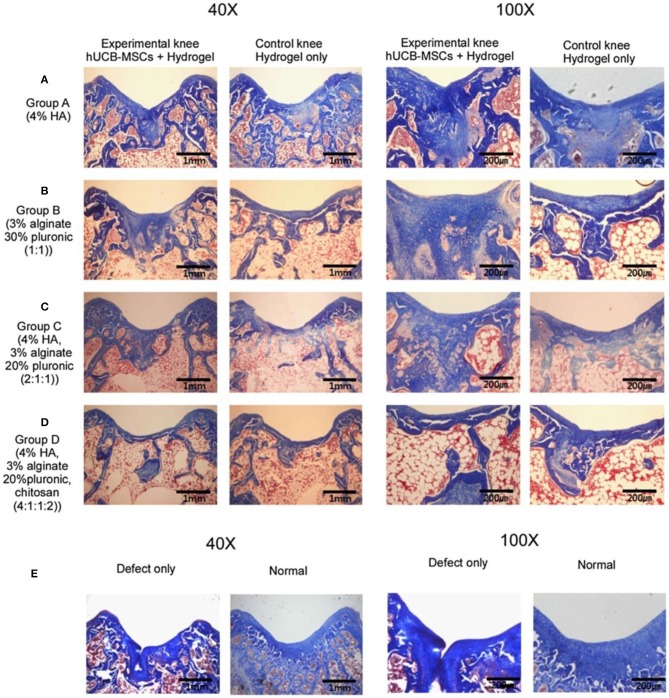
Masson's trichrome staining at 16 weeks post-transplantation. **(A)** Group A with/without hUCB-MSCs + 4% HA hydrogel composites; **(B)** Group B with/without hUCB-MSCs + 3% alginate:30% pluronic (1:1, v/v) composites; **(C)** Group C with/without hUCB-MSCs + 4% HA: 3% alginate: 20% pluronic (2:1:1, v/v) composites; **(D)** Group D with/without hUCB-MSCs + 4% HA: 3% alginate: 20% pluronic: chitosan (4:1:1:2, v/v); **(E)** Defect only and normal cartilage. Reproduced with permission from Chung et al. (2014).

UCB-MSCs have attracted wide interest as a promising source of regenerative medicine cells due to their non-invasive collection, high expansion capacity, availability, and low immunogenicity. These cells have been identified and found phenotypic similarities to BM-MSCs and embryonic stem cells. Chung et al. explored the feasibility and efficacy of repairing articular cartilage using a composite of hUCB-MSCs and four different hydrogels in a rat model. The results showed that group with 4% HA hydrogel can significantly improve cartilage histologically and achieved the cellular arrangement and collagen tissue pattern mimicking adjacent undamaged articular cartilage (Figure 8; Chung et al., 2014). Park et al. demonstrated that treatment with undifferentiated vs. chondrogenic predifferentiated hUCB-MSCs and 4% HA hydrogel resulted in more approving cartilage repair than the control groups with chondro-MSCs in a rat model (Park et al., 2019). From the clinical trial for safety and proof-of-concept with 7 years of extended follow-up, Park et al. reported that the hUCB-MSCs-based HA hydrogels appeared to be safe and effective for cartilage regeneration in osteoarthritic patients (Park et al., 2017).

### Autologous Activated Peripheral Blood Stem Cells (AAPBSCs)

AAPBSCs contain MSCs with chondrocyte precursor potential to initiate hyaline cartilage remodeling (Duff et al., 2003; Turajane et al., 2014). The clinical trial studied by Saw's groups showed that, arthroscopic cartilage can be regenerated with arthroscopic subchondral drilling followed by injecting autologous PBPCs and HA into postoperative intra-articularly (IA) autologous (Saw et al., 2011). The combination of AAPBSC and HA on the autologous cancellous bone scaffold initiated the chondrocyte differentiation. And the addition of platelet-rich plasma (PRP) and human granulocyte colony stimulating factor (hG-CSF) further stimulated the proliferation of cells to the chondrocyte phenotype with enhanced Sox9 transcription. The above processes led to the continuous increase of col-2 and aggrecan mRNA, which ultimately resulted in the histologically confirmed proteoglycan and glycosaminoglycan contents increase in newly formed in transparent cartilage (Turajane et al., 2014). The combination of AAPBSCs with growth factor addition/preservation with HA and arthroscopic microdrilling MCSs can improve cartilage regeneration in early knee disease that failed conservative treatment (Turajane et al., 2013).

## Summary and Perspectives

This paper reviews the latest progress in the design and preparation of stem cell-laden hydrogel for osteochondral tissue engineering applications in terms of engineering hydrogel properties, biomimetic microenvironment, and growth factor delivery. Stem cell-based therapies have recently opened up new opportunities for clinical applications to treat diseases that cannot be effectively treated with conventional chemotherapy. MSCs are isolated from different tissues, such as bone marrow, adipose tissue, placenta, umbilical cord blood, and peripheral blood. Stem cells can sustainably release of therapeutic small molecules that are important for cell survival and tissue regeneration, which has been acknowledged as an essential treatment for effective treatment of various diseases. Despite the considerable potential of these stem cell therapies, the reduced viability of transplanted stem cells after transplantation often leads to unsatisfactory results in *in vivo* studies. The microenvironment of damaged tissue, such as reactive oxygen species and host immune responses, is unfavorable for growth of stem cells. In addition, the absence of cell support signals around damaged tissues can also result in the ultimate death of the transplanted stem cells. Therefore, it is important for the research focusing on the stem cell transplantation in combination with substances supporting cell survival, inducing cell bioactivity, and enhancing cell retention at managed sites. Especially, hydrogels supplying a tissue-like environment have been widely studied as a vehicle for delivering stem cells.

Hydrogel materials afford control over critical regulators of stem cell fate, including matrix mechanics and biochemistry, microscale structure, and cell-cell interactions. With the development of tissue engineering and the regenerative medicine, it is found that tissue regeneration and reconstruction require a multifunctional scaffold to load and delivery tissue-specific cells. In this sense, hydrogel scaffolds are recognized as ideal biomaterials for the tissue engineering of cartilage, bone, skin, heart valves, nerves, tendons, etc., due to their composition, structure, morphology, function, and mechanics are closely similar to the natural tissue extracellular matrix. The hydrogels and 3D architecture scaffolds combined with various bioactive molecules, genes and cells as well as the tunable mechanical properties have capacity to guide and promote the *in vivo* implantation and development of multifunctional engineered tissues. Thus, these hydrogels scaffolds with customized morphologies and suitable mechanical behaviors bring the prospect of cartilage tissue engineering through the tailorable retention, and delivery abilities of cells and growth factors in the injury site.

Chondrocytes can successfully repair focal cartilage, however, the problems including the limited supply of them, long expansion time, and may be differentiated into fibroblasts. On the other hand, stem cells have more application prospects due to their rich in source and potential to differentiate into chondrocytes. Various types of stem cells encapsulated in hydrogels can differentiate into chondrocytes or osteoblasts under the induction of growth factors. Delivery of growth factors via microencapsulation, covalent bond with hydrogel and gene delivery is attractive for the localized release of inducers.

Even though, it should be noted that it is still a major challenge to fully restore cartilage to its original composition, architecture, mechanics, and biofunction. For example, simultaneous achievement of integrating cartilage and subchondral bone regeneration has been a critical challenge in tissue engineering. The difference of structure and modulus in two distinct types of tissues should be carefully considered for overcoming the difficulties in simulating the structures and functions by the hybrid or bi-phase hydrogel scaffolds. Wherein, the part of cartilage repair exhibited highly elastic modulus to bear the pressure and resist the friction to facilitate the extracellular matrix, enhance the chondrogenesis of MSCs, inhibit the hypertrophic differentiation, and contribute to the chondrocyte mineralization. While another part of subchondral bone repair could effectively contribute to the formation of blood vessel network within the hydrogels to facilitate nutrient transports, stimulate osteoblast proliferations and provide great supports for regenerative cartilage. More importantly, integration of the surrounding cartilage and the implants should possess strong interfacial adhesion that can be significantly enhanced for regenerated cartilage. Additionally, smart incorporations of intelligence or self-guided features also played the essential roles in the fabrication and development of a new kind of cell-laden hydrogels to obtain the fully cartilage regeneration in biomedical applications.

It should be also further noted that although there have been some limited clinically approved tissue-engineered products for the clinical trials status quo in recent years, a rapid progress toward more advanced and targeted therapies is still particularly noticed by promoting microfabrication techniques and developing the cellular scaffold-based approaches. It is concluded that an ideal stem cell-laden hydrogel for achieving the cartilage tissue engineering should synchronously possess the following characterizations: (1) biological activity and biomimetic function; (2) mechanical reinforcement; (3) integration of cartilage with bone tissue; and (4) transport of drugs and growth factors. Therefore, the intelligent and hybrid hydrogel scaffolds with complex architectures should be well-fabricated for realizing the customized clinic treatments. And the corresponding researches on the mechanical and biological behaviors of hydrogel scaffolds should also be emphasized to ensure the powerful tissue interactions, resorption and hierarchical architecture for enabling the tissue engineering implants. With this understanding, the future work should forcefully focus on identifying the secondary, tertiary and higher order architectures of the hybrid hydrogels, quantifying their composition, morphology and function, characterizing their binding pockets and interactions with cell surface receptors and finally turning them into a clinically tissue engineering biomaterial for effective cartilage tissue engineering. In this sense, in the future we should establish such a methodology or criteria on the design and development of final biological tissue engineering products for regenerative medicine, which makes the cell-laden hydrogel satisfy more advantages on adjustable structure, better strength, adequate immune response, adhesive interfacial binding force and good biodegradability for enabling the real applications in human patients.

We are strongly convinced that with the help of continuous developments of cell-laden hydrogels and exquisite adjustment of their physicochemical and mechanical properties for effective osteochondral tissue engineering, more advanced multi-responsive histological engineering products with optimized architectures and functions will be eventually created to obtain the greater manipulation and higher availability for various biomedical applications. The compositions, structures and mechanical properties of newly responsive hydrogels are hopefully to be continually developed, and thus we will further obtain smart biomaterials with topological complexity for tissue engineering of regenerative medicine.

## Author Contributions

BX and JY contributed equally to this review paper. XW and J-KY conceived and designed the content of the paper. BX, JY, F-ZY, J-YZ, Y-RC, and B-SF collected the researched literatures. J-YZ arranged the outline of collected documents. BX and JY wrote the article. DJ and W-BJ made important suggestions and helped revising the paper. All authors reviewed and commented on the entire manuscript.

### Conflict of Interest

The authors declare that the research was conducted in the absence of any commercial or financial relationships that could be construed as a potential conflict of interest.
